# Supplementary material for: Co-occupancy identifies transcription factor co-operation for axon growth
Source: Nat Commun. 2021 May 5;12:2555. doi: 10.1038/s41467-021-22828-3 (PMC8099911; doi:10.1038/s41467-021-22828-3)
Supplement: Supplementary file 1 — Supplementary Information [file 41467_2021_22828_MOESM1_ESM.pdf]

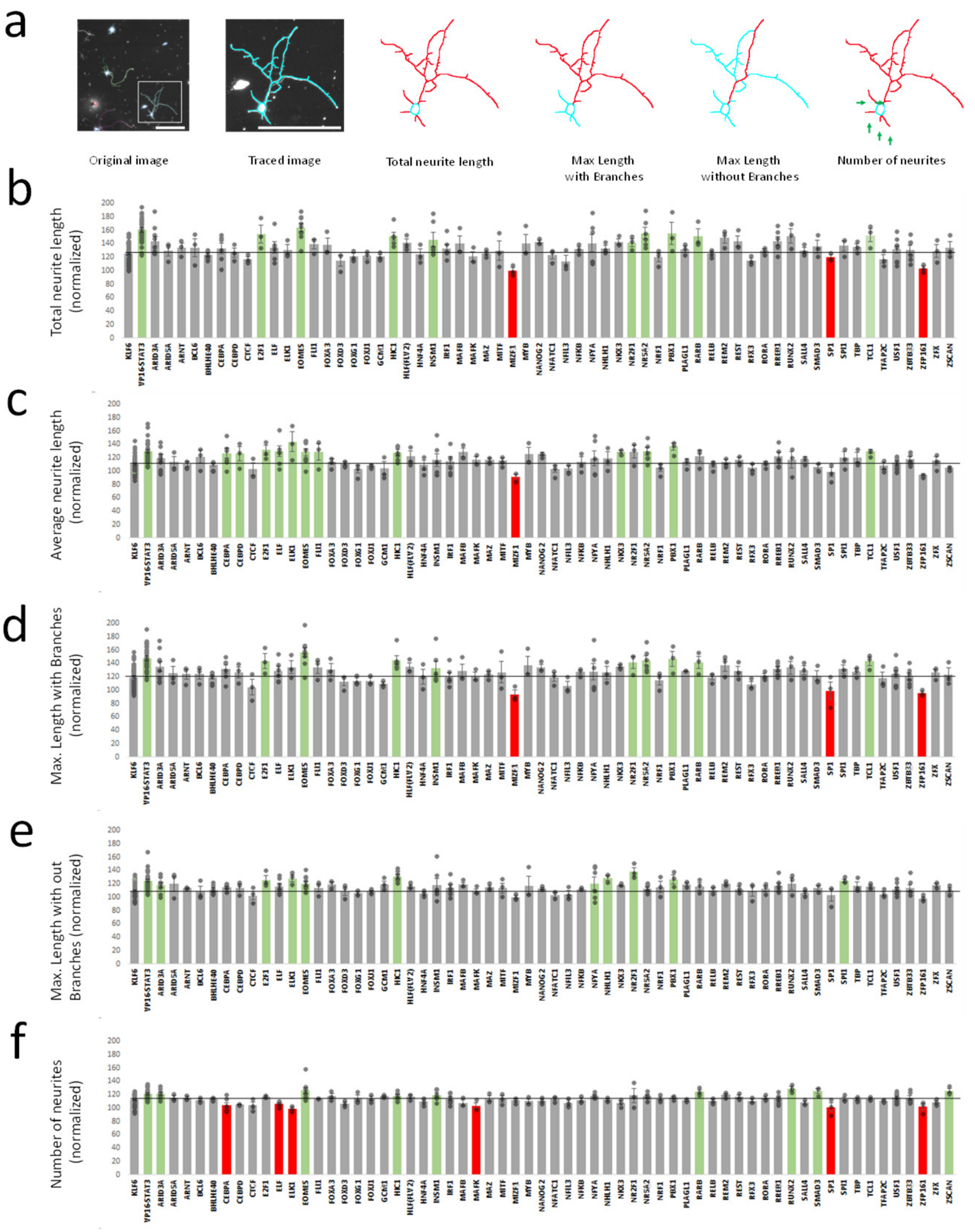

Supplementary Fig.1 | Expanded morphological screening data. (a) shows a transfected cortical neuron and tracing by cellomics, followed by schematic depictions of morphological parameters. (b-f) show the effect of forced expression of Klf6 alone, Klf6 with Vp16-Stat3, or Klf6 with candidate TFs. In all cases measurements are normalized to those of within-plate control plasmids. Green bars indicate significant increases, and red bars indicate significant reductions compared to the values of Klf6 alone ($p<.05$, 1-way ANOVA with post-hoc Fishers LSD). Experiments were repeated three time sindependently with similar results , with a minimum of 150 individual cells analyzed in each;full details regarding the number of analyzed cells and exact p values are available in Supplementary Data 2. Error bars indicate SEM. Scale bars are 100 μm. Source data are provided as a Source Data file.

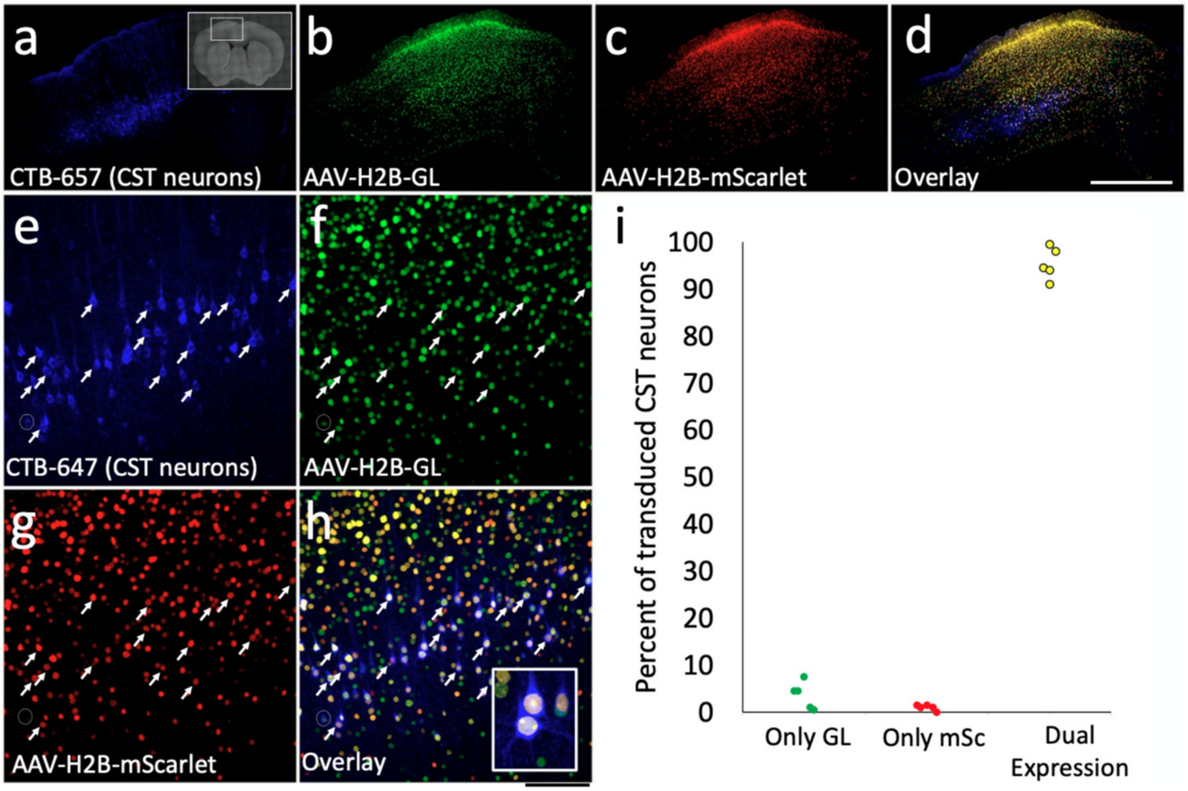

Supplementary Fig. 2. Co-injection of AAV2-Retro drives effective co-expression of transgenes in corticospinal tract neurons. Adult mice received cortical injection of mixed AAV2-Retro-H2B-EGFP and AAV-H2B-mScarlet, followed two weeks later by injection of CTB-647 to cervical spinal cord. Animals were perfused three days later, and transverse sections of cortex examined by confocal microscopy. (a-d) show the distribution of CST neurons and AAV-expressed fluorophores. (e-h) show higher magnification views, illustrating efficient co-expression of both fluorophores in CST neurons (arrows). The circle indicates a rare instance of a CST neuron expressing EGFP (green, F) but not mScarlet (red, G). (I) quantifies the percent of transduced CST neurons that show single expression of either fluorophore or dual expression of both: 95.4% (± 1.51 SEM) of CST neurons were dually transfected. n=200 CST neurons scored from each of 5 mice. Scale bars are 1 mm (a-d) and 0.1mm (e-h). Source data are provided as a Source Data file.

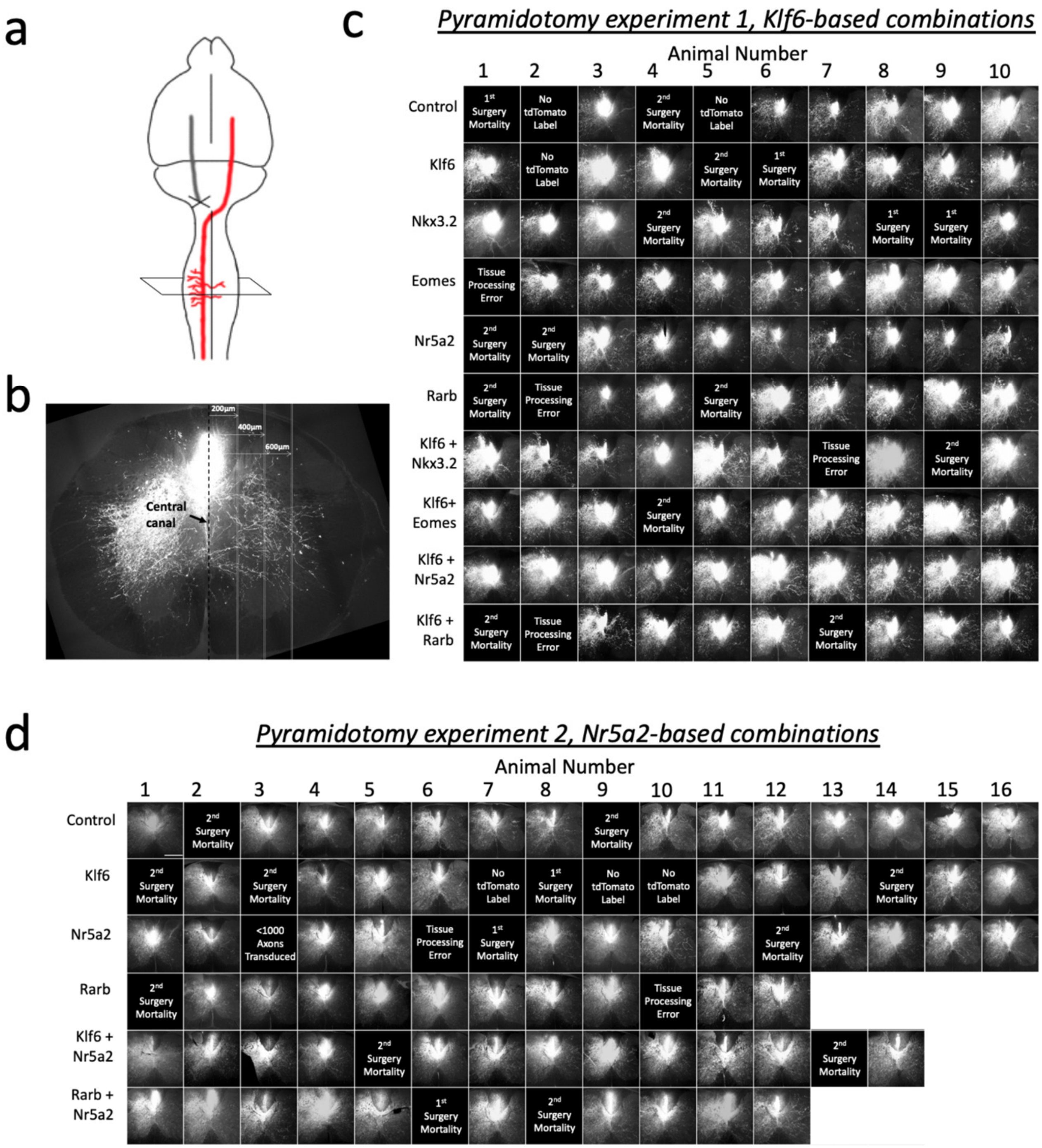

Supplementary Fig. 3 | Summary of *in vivo CST* cross-midline growth after single or combinatorial expression of candidate TFs (a) illustrates the unilateral pyramidotomy injury and indicates the approximate location of images. (b) shows a transverse section of spinal cord, eight weeks after pyramidotomy, with CST axons labeled by tdTomato. Vertical lines show the sampling regions in which axonal profiles were counted at 200, 400, and 600um from the midline. (c,d) provide example images from all animals in the two *in vivo* experiments; where images are missing, the reason for exclusion is indicated.

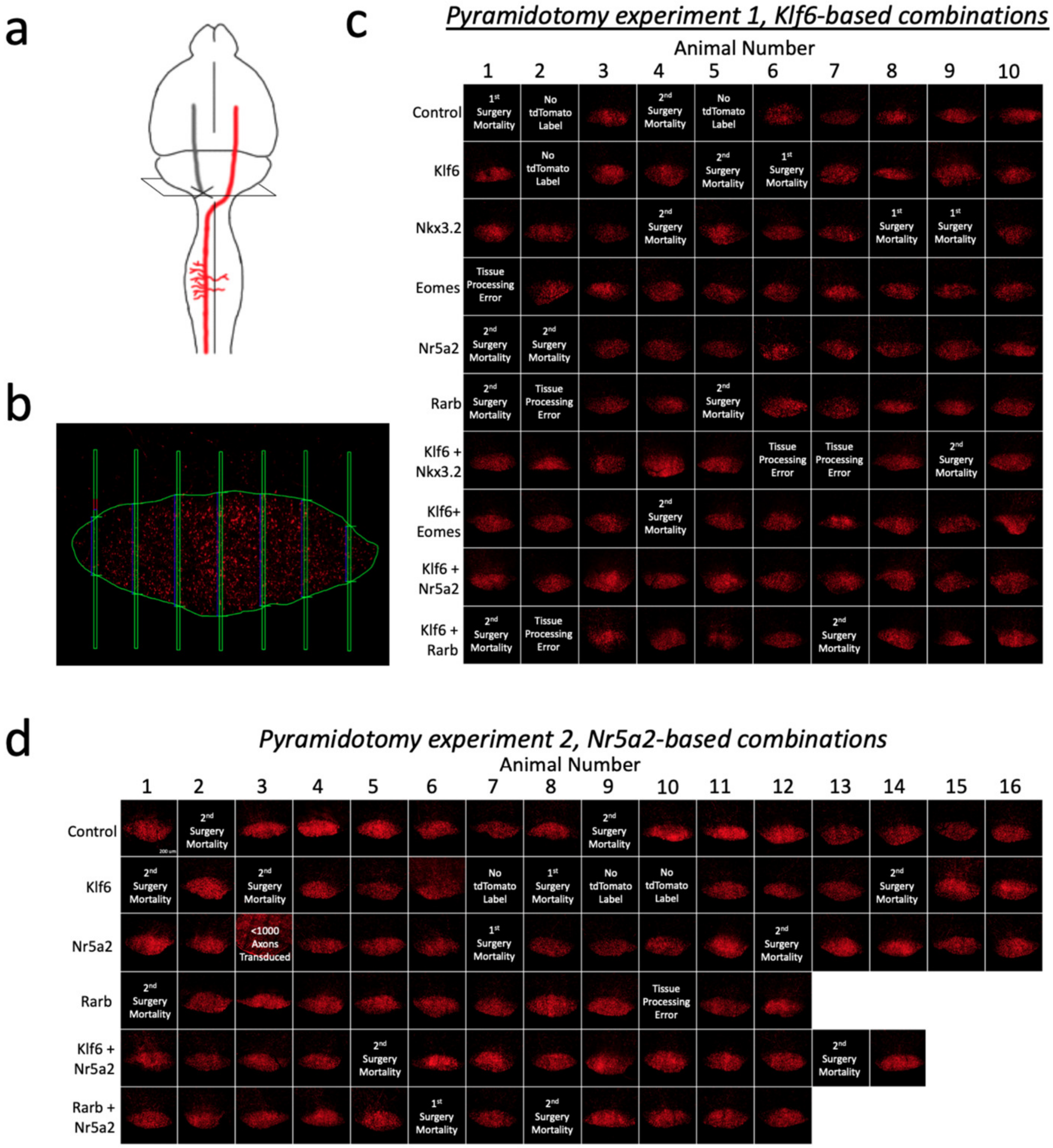

Supplementary Fig. 4 | Summary of tdTomato label in CST axons in the medulla, used to normalize CST counts in the spinal cord (a) illustrates the unilateral pyramidotomy injury and indicates the approximate location of images. (b) shows a transverse section of an example medullary pyramid, nine weeks after cortical injection with AAV-TF treatment and AAV-tdTomato tracer, in which CST axons appear as red (tdTomato+) puncta as they intersect the plane of the section. The pyramid is outlined, and vertical boxes indicate the regions in which each individual axon was counted. Total axon numbers were estimated by multiplying axon counts by the total sampling area, divided by total medullary area. (c,d) provide example images from all animals in two *in vivo* experiments; where images are missing, the reason for exclusion is indicated.

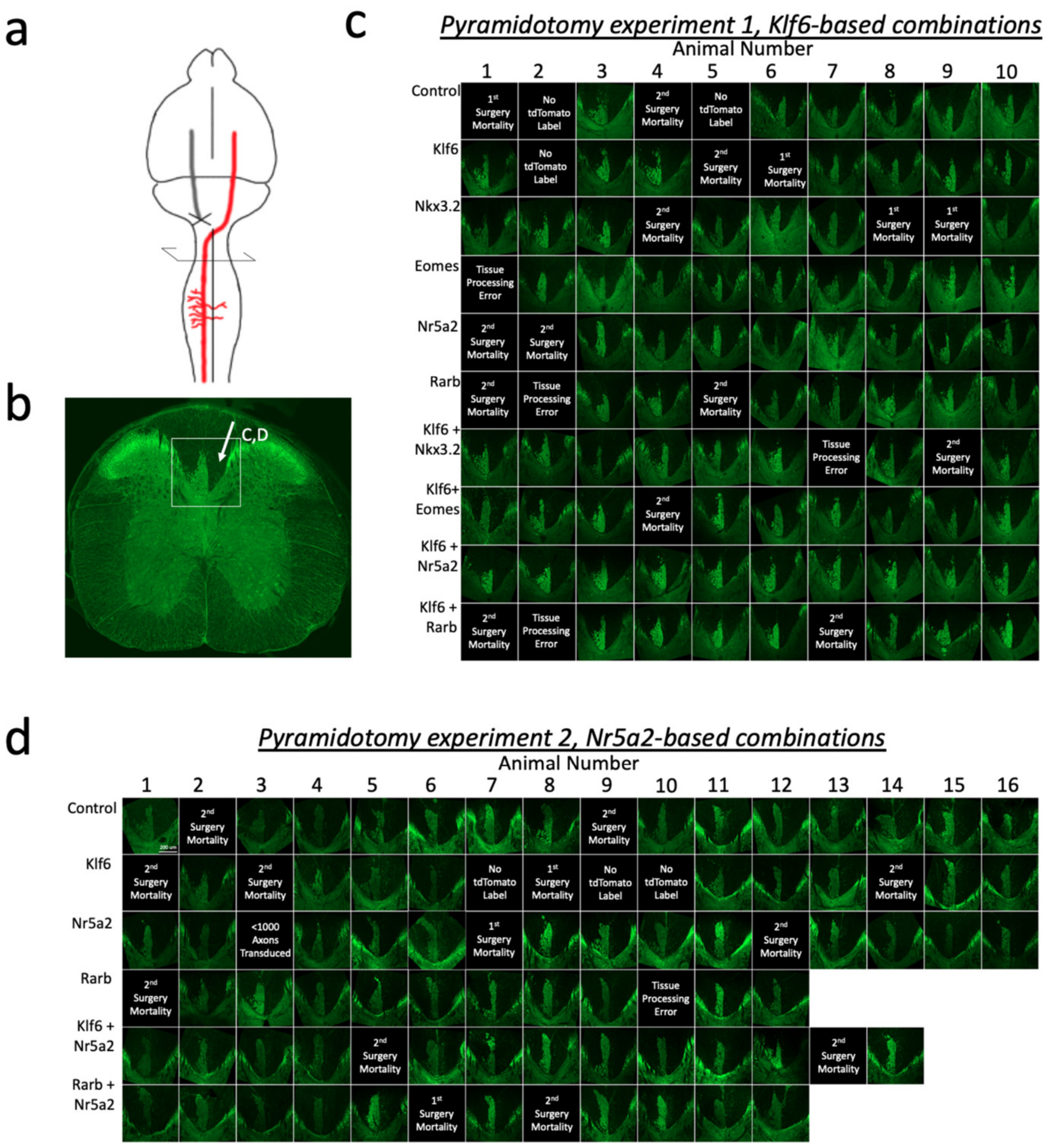

Supplementary Fig. 5 | Summary of PKCγ signal in cervical spinal cord, used to verify unilateral ablation of the CST (a) illustrates the unilateral pyramidotomy injury and indicates the approximate location of images. (b) shows a transverse section of cervical spinal cord, eight weeks after pyramidotomy injury, with PKCγ signal (green) readily detectable in the intact but not transected CST (white arrow). (c,d) provide example images of the dorsal columns after PKCγ staining from all animals in two *in vivo* experiments; where images are missing, the reason for exclusion is indicated.

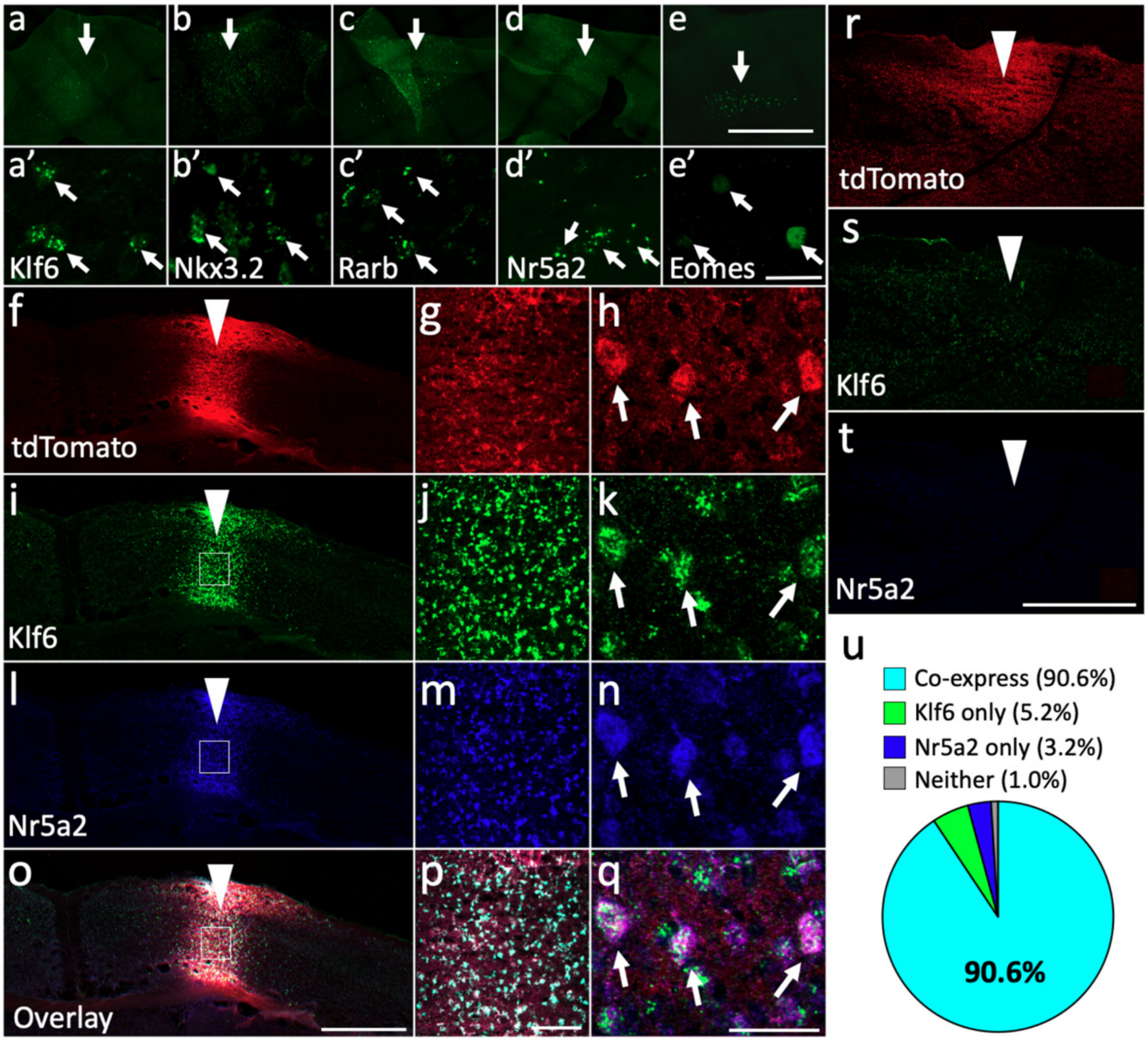

Supplementary Fig. 6 | RNAscope and immunohistochemistry confirm expression of candidate transcription factors (a-e) show coronal section of adult mouse cortex, eight weeks after cortical injection of AAV expressing candidate transcription factors, with arrows marking the site of injection. RNAscope or appropriate antibodies were used to probe for expression. RNAscope signal from probes directed against the expressed TFs is present at the injection site, which at higher magnification (a'-d') shows the characteristic punctate detection of transcripts. e and e' show detection of Eomes by immunohistochemistry. (f-u) Adult mice received cortical injection of AAV-Klf6, AAV-Nr5a2, and AAV-tdTomato at 1.5:1.5:1 ratio, the same used in axon growth experiments. Two weeks later cortices were examined by fluorescent in situ hybridization (RNAscope) to visualize expression of Klf6 and Nr5a2. (f-h) show tdTomato at the site of viral injection, (i-k) show Klf6 expression, (l-n) show Nr5a2, and (o-q) show the overlay. Note that tissue distant from the injection site displays very low levels of Klf6 and Nr5a2 detection, while virally-expressed transgenes are readily detected at the site of injection. (r-t) show a cortex that received AAV-tdTomato and AAV-Cre control (arrowhead), with low detection of endogenous Klf6 (s) and Nr5a2 (t) transcripts. (u) tdTomato+ cells were classified according to dual, single, or no expression of Klf6 and Nr5a2 transcripts; more than 90% of tdTomato+ cells expressed both transcripts. n = 475 cells analyzed from three animals. Scale bars are 1mm (a-e, f, i, l, o, r-t), 50 µm (a'-e'), and 100 µm (g, h,j, k, m, n, p, q). Source data are provided as a Source Data file.

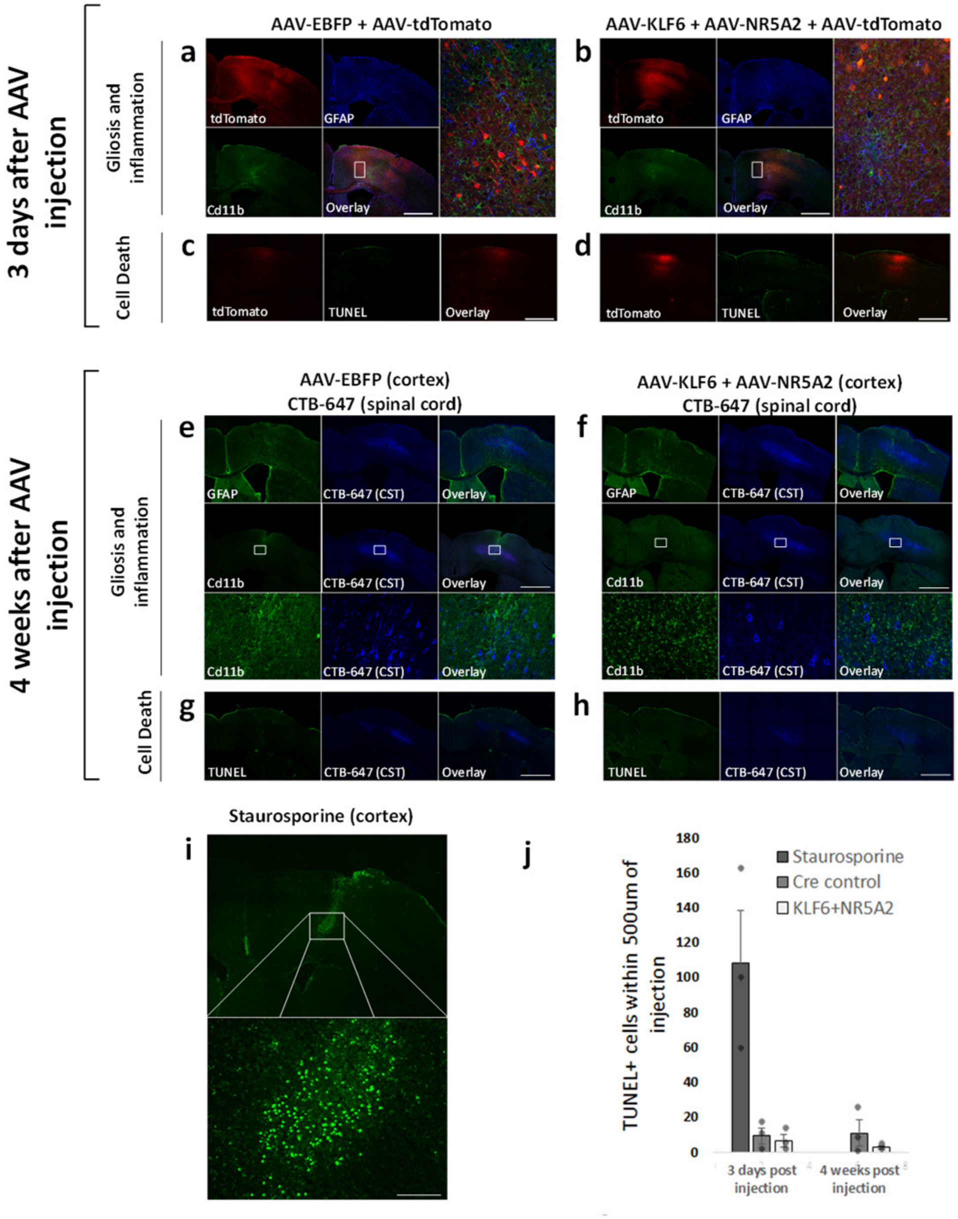

Supplementary Fig. 7. Cortical injection of AAV-Klf6 and AAV-Nr5a2 does not elevate gliosis, inflammation, or cell death. (a-d) Adult mice received cortical injection of AAV-tdTomato with AAV-Cre or combined AAV-Klf6/AAV-Nr5a2 (KN). Three days later, animals were sacrificed, and coronal sections of cortex examined for gliosis (GFAP), inflammation (CD11b), or cell death (TUNEL). Control and KN-injected animals showed similar levels of GFAP and CD11b near the site of injection (a, b) and minimal cell death (c,d). (e-h) Animals received cortical injection of AAV-Cre control or AAV-K/N and cervical injection of CTB-647 to label CST neurons. Four weeks later, coronal sections of cortex were stained for GFAP, CD11B, or TUNEL reactivity. CST neurons were apparent in the vicinity of the injection, and minimal levels of GFAP and CD11B persisted. (g, h) TUNEL signal was rare in both treatments, and never co-localized with CST neurons. (i) Shows numerous TUNEL-positive cells (green) 2 days after Staurosporine injection, confirming assay sensitivity. (j) quantifies the average number of TUNEL+ cells located within 500µm of the injection track. Compared to staurosporine, AAV- Cre control and AAV-KLF6/Nr5a2 produce TUNEL+ cells at low numbers that do not statistically differ (Staurosporine vs. Cre p=0.0041, Staurosporine vs. KN p=0.0033, Cre vs. KN p=0.999, 1-way ANOVA with post-hoc Dunnett's). n=3 animals in each group, three replicate sections per animal. Scale bars are 1mm (a-h) or 100 µm (i). Source data are provided as a Source Data file.

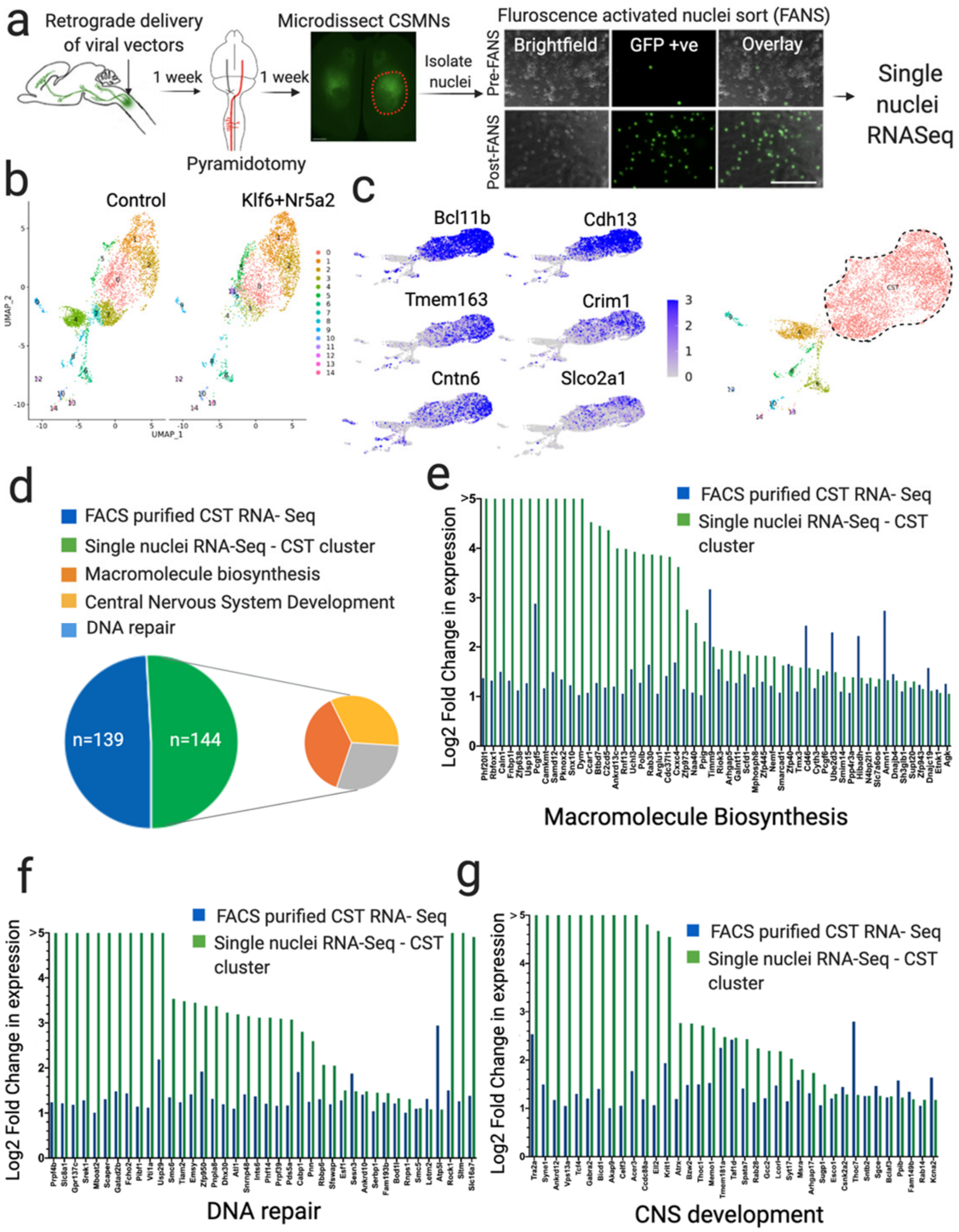

Supplementary Fig. 8 | Single nuclei RNASeq analyses validate transcriptional changes following combined KLF6/Nr5a2 gene treatment (a) Overview of sample collection for Single nuclei RNA-Seq analysis. (b) UMAP visualization of 3383 nuclei (Control) and 3038 nuclei (KLF6+Nr5a2 treated) that passed QC filtering (see methods) confirms qualitative concordance across groups (c) Expression of key marker genes delineated nuclei clusters specific to Corticospinal tract neurons (d) Bioinformatic analyses confirmed ~ 50% overlap in Klf6/Nr5a2 responsive target genes between the two independent RNA-Seq approaches. Regulatory network analysis of genes upregulated after combined Klf6/Nr5a2 overexpression confirmed sub-networks enriched for functional categories relevant to axon growth (e-g) Agreement of log2 Fold change in expression of functionally distinct Klf6/Nr5a2 responsive target genes between the two independent RNA-Seq approaches. Differential testing – Non-parametric Wilcoxon rank sum test (SEURAT v3).n=3 animals/rep and 2 reps/treatment. Each experiment in panel b was repeated twice independently with similar results (Supplementary Data 4). Scale bar (a) = 100μm. Source data are provided as a Source Data file.

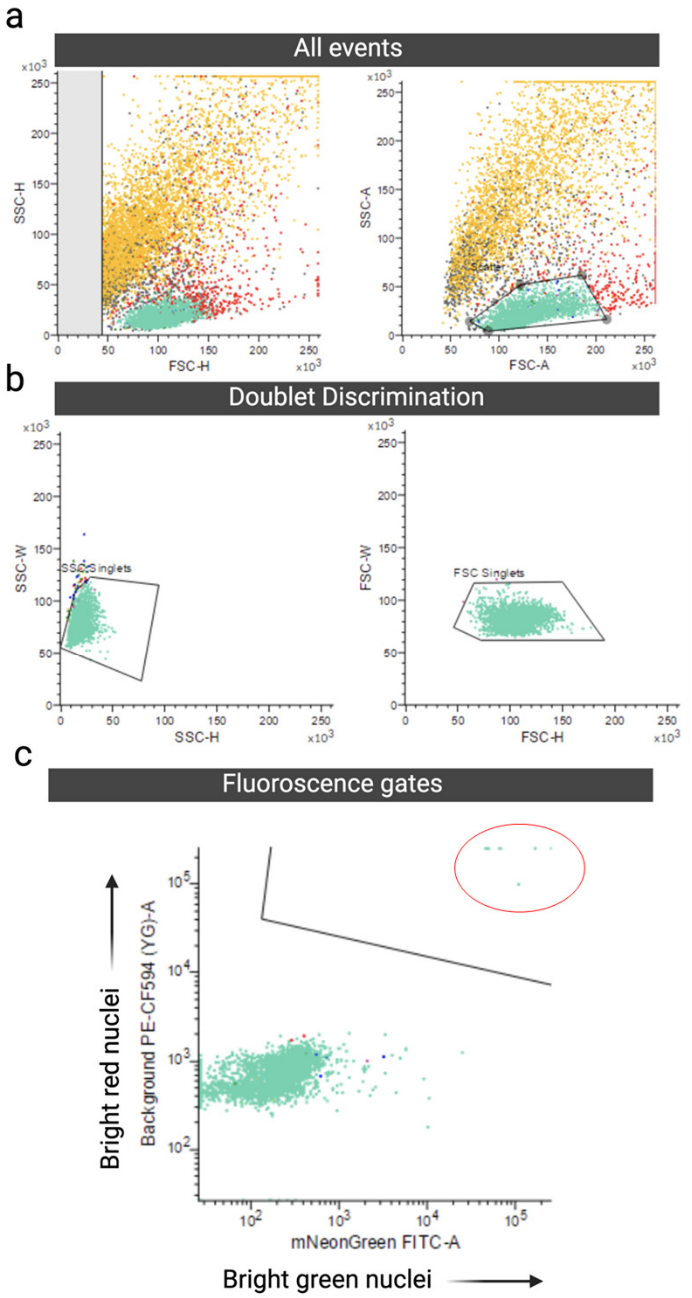

Supplementary Fig. 9 | Fluorescence activated nuclei sorting (FANS) isolates intact labeled nuclei for transcriptional profiling. Dissociated nuclei were flow-sorted on a BD FACS Melody using an 80um nozzle and a sequential gating strategy (Sort type: Purity). (a) Nuclei were first gated by SSH-H vs FSC-H and SSC-A vs FSC-A to exclude sheared fragments/debris from intact nuclei. (b) Doublets were excluded by plotting FSC, SSC-area against height and gating against intact nuclei with increased area. (c) Intact labeled nuclei were isolated by plotting mNeon+ (FITC) signal against a background of mScarlet+ (PE-CF594) signal and manually gating for nuclei with high FITC and PE-CF594 signal to only collect the brightest nuclei ((log10 scale > 10 to the power 4, red circle). Nuclei were collected to a goal of approximately 30,000 events for RNA-Seq and 4000 events for single-nuclei RNA-Seq.
