# Supplementary material for: Co-occupancy identifies transcription factor co-operation for axon growth
Source: Nat Commun. 2021 May 5;12:2555. doi: 10.1038/s41467-021-22828-3 (PMC8099911; doi:10.1038/s41467-021-22828-3)
Supplement: Supplementary file 3 — Description of Additional Supplementary Files [file 41467_2021_22828_MOESM3_ESM.pdf]

Description of Additional Supplementary Files

Supplementary Data 1: List of developmentally downregulated genes and GO terms along with relevant enhancers

Supplementary Data 2: Summary of *in vitro* screening experiments

Supplementary Data 3: Summary of viral titers used in the study

Supplementary Data 4: List of differentially expressed genes upon Klf6, Nr5a2 and combined Klf6/Nr5a2 treatment following RNA-Seq
