# Supplementary material for: Co-occupancy identifies transcription factor co-operation for axon growth
Source: Nat Commun. 2021 May 5;12:2555. doi: 10.1038/s41467-021-22828-3 (PMC8099911; doi:10.1038/s41467-021-22828-3)
Supplement: Supplementary file 8 — Reporting summary [file 41467_2021_22828_MOESM8_ESM.pdf]

nature research

Corresponding author(s): Ishwariya Venkatesh and Murray Blackmore

Last updated by author(s): March 9, 2021

Reporting Summary

Nature Research wishes to improve the reproducibility of the work that we publish. This form provides structure for consistency and transparency in reporting. For further information on Nature Research policies, see our Editorial Policies and the Editorial Policy Checklist.

Statistics

For all statistical analyses, confirm that the following items are present in the figure legend, table legend, main text, or Methods section.

n/a	Confirmed	
☐	☒	The exact sample size (n) for each experimental group/condition, given as a discrete number and unit of measurement
☐	☒	A statement on whether measurements were taken from distinct samples or whether the same sample was measured repeatedly
☐	☒	The statistical test(s) used AND whether they are one- or two-sided<br>*Only common tests should be described solely by name; describe more complex techniques in the Methods section.*
☒	☐	A description of all covariates tested
☐	☒	A description of any assumptions or corrections, such as tests of normality and adjustment for multiple comparisons
☐	☒	A full description of the statistical parameters including central tendency (e.g. means) or other basic estimates (e.g. regression coefficient) AND variation (e.g. standard deviation) or associated estimates of uncertainty (e.g. confidence intervals)
☐	☒	For null hypothesis testing, the test statistic (e.g. F, t, r) with confidence intervals, effect sizes, degrees of freedom and P value noted<br>*Give P values as exact values whenever suitable.*
☒	☐	For Bayesian analysis, information on the choice of priors and Markov chain Monte Carlo settings
☒	☐	For hierarchical and complex designs, identification of the appropriate level for tests and full reporting of outcomes
☒	☐	Estimates of effect sizes (e.g. Cohen's d, Pearson's r), indicating how they were calculated

Our web collection on statistics for biologists contains articles on many of the points above.

Software and code

Policy information about availability of computer code

Data collection	no software was used for data collection under data collection
Data analysis	There was no custom code development and all software used in data analyses are previously published, open-access and have been cited under the relevant methods section. Links to relevant software repositories/documentation is listed here -<br>WGCNA - https://horvath.genetics.ucla.edu/html/CoexpressionNetwork/Rpackages/WGCNA/ ;<br>RNA-Seq analyses - https://github.com/ENCODE-DCC/rna-seq-pipeline;<br>EdgeR- https://www.bioconductor.org/packages/release/bioc/html/edgeR.html;<br>Activity-by-contact (ABC) - https://github.com/broadinstitute/ABC-Enhancer-Gene-Prediction;<br>oPOSSUM 3.0- http://opossum.cisreg.ca/oPOSSUM3/ ;<br>Cytoscape - ClueGO - http://www.ici.upmc.fr/cluego/cluegoDocumentation.shtml ;<br>Harmonizome - https://amp.pharm.mssm.edu/Harmonizome/;<br>iRegulon - http://iregulon.aertslab.org/<br>Cellranger - https://github.com/10XGenomics/cellranger<br>SEURAT - https://github.com/satijalab/seurat<br>FACSChorus - https://www.bdbiosciences.com/en-us/instruments/research-instruments/research-software/flow-cytometry-acquisition/facschorus-software<br>DAVID- https://david.ncifcrf.gov/summary.jsp

For manuscripts utilizing custom algorithms or software that are central to the research but not yet described in published literature, software must be made available to editors and reviewers. We strongly encourage code deposition in a community repository (e.g. GitHub). See the Nature Research guidelines for submitting code & software for further information.

Data

Policy information about availability of data

All manuscripts must include a data availability statement. This statement should provide the following information, where applicable:

- Accession codes, unique identifiers, or web links for publicly available datasets
- A list of figures that have associated raw data
- A description of any restrictions on data availability

There are no restrictions on data availability. All the datasets generated and analyzed during the current study are available in the NCBI repository (PRJNA630017 - https://www.ncbi.nlm.nih.gov/Traces/study/?acc=PRJNA630017&o=acc_s%3Aa). Source data are provided with this paper. The following publicly available datasets were also analyzed in this study-ATAC-Seq (ENCSR310MLB; https://www.encodeproject.org/experiments/ENCSR310MLB/, ENCSR836PUC; https://www.encodeproject.org/experiments/ENCSR836PUC/), H3K27Ac histone ChIP-seq (ENCSR094TTT; https://www.encodeproject.org/files/ENCFF195BGJ/, ENCSR428OEK; https://www.encodeproject.org/experiments/ENCSR428OEK/), RNA-seq (ENCSR362AIZ; https://www.encodeproject.org/experiments/ENCSR362AIZ/ , ENCSR080EVZ; https://www.encodeproject.org/experiments/ENCSR080EVZ/) and HiC (GSE96107; https://www.ncbi.nlm.nih.gov/geo/query/acc.cgi?acc=GSE96107).

Field-specific reporting

Please select the one below that is the best fit for your research. If you are not sure, read the appropriate sections before making your selection.

☒ Life sciences ☐ Behavioural & social sciences ☐ Ecological, evolutionary & environmental sciences

For a reference copy of the document with all sections, see nature.com/documents/nr-reporting-summary-flat.pdf

Life sciences study design

All studies must disclose on these points even when the disclosure is negative.

Sample size	For in vivo experiments, animal numbers were determined by power analysis with G*Power software (!=0.05, Power=0.8) using historical estimates of variability and adjusted for prior rates of animal attrition.
Data exclusions	Animals that met any one of the exclusion criteria listed below were excluded from pyramidotomy experiments (sup figs 2-5)-<br>1. Animals with less than 80% decrease in PKC" in the affected CST.<br>2. <1000 transduced axons<br>3. No detectable Tdtomato signal (required to visualize CST tract)<br>4. Errors in tissue processing
Replication	For all cell culture experiments, neurite length from a minimum of 150 cells per treatment was averaged, and each experiment was repeated a minimum of three times on separate days. All RNA-Seq experiments were designed such that there are at least two replicates per treatment and we confirmed high replicate concordance before proceeding with downstream analyses. In vivo experiments were performed in two separate batches on separate days with 6-7 animals per treatment group per batch. We have provided example images from all animals in two in vivo experiments; where images are missing, the reason for exclusion is indicated. All attempts at replication were successful.
Randomization	In vivo experiments were designed such that animals were randomized prior to viral treatment, with each surgical day, including equal numbers from each group. Next gen sequencing libraries were randomized by randomly choosing control and treatment libraries that were sequenced on the same lanes. In vitro screening experiments were randomized by ensuring that control and treatment wells were spread across different plate positions for replicates, chosen randomly prior to high-content screening.
Blinding	In vivo experiments were performed in a double-blind fashion, with non-involved lab personnel maintaining blinding keys. Counting of digital images was performed by three blinded observers, with final values reflecting the average. Blinding was not possible during generation of NGS libraries due to the need to track control vs treated libraries separately.

Reporting for specific materials, systems and methods

We require information from authors about some types of materials, experimental systems and methods used in many studies. Here, indicate whether each material, system or method listed is relevant to your study. If you are not sure if a list item applies to your research, read the appropriate section before selecting a response.

Materials & experimental systems

n/a	Involved in the study
☐	☒ Antibodies
☒	☐ Eukaryotic cell lines
☒	☐ Palaeontology and archaeology
☐	☒ Animals and other organisms
☒	☐ Human research participants
☒	☐ Clinical data
☒	☐ Dual use research of concern

Methods

n/a	Involved in the study
☒	☐ ChIP-seq
☐	☒ Flow cytometry
☒	☐ MRI-based neuroimaging

Antibodies

Antibodies used	PKCγ (SC C-19, Santa Cruz, Dallas, TX, 1:500, RRID: AB_632234), GFAP (DAKO, Z0334 1:500, RRID:AB_10013482), or Cd11b (Invitrogen 14-01120-82 1:500, RRID:AB_2536484) , Eomes (ab23345, RRID:AB_778267,1:500) , Alexa Fluor-conjugated secondary antibodies (R37116,R37117,Thermofisher, Waltham, MA, 1:500.)
Validation	PKC" antibody was validated by the manufacturer Santa Cruz and antibody specificity was confirmed by the presence of readily detectable signal only in intact CST and not in the transected portion, and this is highly consistent and observed across all animals in the In vivo experiment (supp fig 3). EOMES antibody was validated by the manufacturer Abcam by demonstrating specific immunohistochemistry signal on adult mouse forebrain tissue (SVZ) and E14 mouse forebrain tissue (https://www.abcam.com/tbr2--eomes-antibody-ab23345.html#description_images_1). GFAP antibody specificity was confirmed by enhanced signal surrounding injury site, indicative of gliosis and by the manufacturer by demonstrating specificity by Immunoprecipitation (https://www.agilent.com/en/product/immunohistochemistry/antibodies-controls/primary-antibodies/glial-fibrillary-acidic-protein-(dako-omnis)-76214#specifications CD11b specificity was validated by the manufacturer Invitrogen by demonstrating specific signal on mouse bone marrow tissue (https://www.thermofisher.com/antibody/product/CD11b-Antibody-clone-M1-70-Monoclonal/14-0112-82).

Animals and other organisms

Policy information about studies involving animals; ARRIVE guidelines recommended for reporting animal research

Laboratory animals	All animal testing and research was carried out in compliance with ethical regulations laid out by the National Institutes of Health guide for the care and use of animals and all experimental protocols involving animals were approved by the Institutional Animal Care and Use Safety committee (IACUC) at Marquette University (protocol number AR-309, AR-314). Mice were bred and raised under a 24-h light-dark cycle with 12 h of light and 12 h of darkness. Ambient temperature was maintained at 22 °C ± 2 °C and humidity between 40-60%. Cortical neurons for screening experiments were derived from early postnatal (P5-P7) Sprague Dawley rat pups (Harlan) of mixed sex.In vivo pyramidotomy experiments were performed on adult female mice (8 weeks of age - C57/BL6). For RNA-Seq data generation,equal number of male and female adult mice (C57/BL6- 8 weeks) were used.
Wild animals	n/a
Field-collected samples	n/a
Ethics oversight	n/a

Note that full information on the approval of the study protocol must also be provided in the manuscript.

Flow Cytometry

Plots

Confirm that:

☒ The axis labels state the marker and fluorochrome used (e.g. CD4-FITC).

☒ The axis scales are clearly visible. Include numbers along axes only for bottom left plot of group (a 'group' is an analysis of identical markers).

☒ All plots are contour plots with outliers or pseudocolor plots.

☒ A numerical value for number of cells or percentage (with statistics) is provided.

Methodology

Sample preparation	Adult mice received retrograde injections of viral vectors for Ctrl treatment, Klf6 alone treatment, or combined Klf6+ Nr5a2 treatment. One week later, animals were challenged with pyramidotomy injuries. One week post-injury, animals were euthanized, and the motor cortices were dissected. Dissected cortices were minced finely using razor blades and transferred to pre-chilled 15 ml Dounce homogenizer filled with 3 ml Nuclear release buffer (320mM Sucrose, 5mM Cacl2, 3mM MgCl2,

	10mM Tris-HCL, 0.3% Igepal). Tissue was dounced 15X while on ice and filtered sequentially via a 50um, 20um filter, and used as input for flow cytometry
Instrument	BD FACS Melody
Software	BD FACSChorus
Cell population abundance	Dissociated nuclei were flow-sorted on a BD FACS Melody using an 80um nozzle to a goal of approximately 30,000 events. Sorted nuclei were visualized under a fluorescent microscope to confirm purity (>90% mNeongreen+ nuclei).
Gating strategy	All events were gated preliminarily using FSC/SSC - Area to exclude sheared fragments/debris from intact nuclei. Next, doublets were excluded by plotting FSC- area against FSC-height and gating against nuclei with increased area. Finally, labeled nuclei were isolated by plotting mNeon+ (FITC signal) against a background of PE-CF594 and manually gating for nuclei with high FITC signal (log10 scale > 10 to the power 4). Sorting strategy from a representative sort is shown on Supplementary Fig 9.

☒ Tick this box to confirm that a figure exemplifying the gating strategy is provided in the Supplementary Information.
